# Immunity, parasites, genetics and sex hormones: contributors to mild inflammatory responses in COVID-19?

**DOI:** 10.11604/pamj.supp.2020.35.2.23267

**Published:** 2020-05-15

**Authors:** Samuel Munalula Munjita, Mulemba Samutela, Kunda Ndashe, Sody Mweetwa Munsaka

**Affiliations:** 1 Department of Biomedical Sciences, School of Health Sciences, University of Zambia, Lusaka, Zambia; 2 Department of Disease Control, School of Veterinary Medicine, University of Zambia, Lusaka, Zambia; 3 Department of Paraclinical studies, School of Veterinary Medicine, University of Zambia, Lusaka, Zambia; 4 Department of Environmental Health, Faculty of Health Sciences, Lusaka Apex Medical University, Lusaka, Zambia

**Keywords:** COVID-19, mild symptoms, cytokines, inflammation, immunity, parasites, genetics, hormones

## Abstract

The Coronavirus disease 2019 (COVID-19) pandemic has killed over two hundred thousand people by end of April, 2020. America and Europe top in deaths from COVID-19 whereas the numbers are lower in Africa for unclear reasons. Emerging evidence suggests the role of hyperactive immune responses characterised by high pro-inflammatory cytokines in severe cases of COVID-19 and deaths. In this perspective, we explore the possible factors that may contribute to mild inflammatory responses in some cases of COVID-19 by focusing on immune education, parasites, sex hormones and chronic diseases, as well as genetic tolerance. To build our perspective, evidence is also extracted from wild rodents due to their multi-tasking immune responses as a result of constant exposure to pathogens.

## Essay

Immune responses characterised by high levels of cytokines have been associated with poor prognosis for COVID-19 [1-3]. People with compromised immune responses due to old age and chronic diseases are considered to be at higher risk of the disease [4,5]. Conversely, children (except infants) and teens, often known to have immature immune systems, generally have mild to moderate symptoms for COVID-19 [5,6]. Meanwhile relatively low death rates in some regions with a high infectious disease burden and a relatively young population have been observed [7]. Although it is too early to make meaningful conclusions, immature and poorly functional Angiotensin-converting enzyme II (ACE2) receptors have been suggested to play a role in mild COVID cases among children [8,9]. Regardless, we think that other contributing factors include cross-reactive antibodies from seasonal coronavirus infections, genetic tolerance, sex hormones, and frequent contact with diverse pathogens and antigens. An immune system continuously exposed to pathogens may be educated to respond favourably to avoid immune-mediated pathology as observed elsewhere [10]. At the centre of such a system are mild to moderate cytokine responses. Although not the mainstay of this perspective, we accept that previous vaccination with Bacillus Calmette-Guerin (BCG) may have non-specific protective effects against SARS-CoV-2 infections [11,12]. In this perspective, we analyse the factors and mechanisms that may contribute to mild or asymptomatic cases of SARS-CoV-2 infections. We base some of the assumptions and perspectives on immune responses in wild rodents which constantly encounter various pathogens.

**Inflammation in COVID-19:** free living rodents in the wild are frequently exposed tovarious infectious agents. Their immune systems may be experienced to produce aggressive immune responses, but have evolved to respond mildly [10,13]. This may be a homeostatic mechanism to reduce immune-mediated injury [13]. Although this does not apply to all viruses [14-16], lessons can be drawn to understand the possible mechanisms that may lead to asymptomatic COVID-19 or severe cases. Generally, immune responses of rodents in the wild are characterised by highly elevated Th2 responses with depressed Th1 cytokine responses [17,18]. In contrast, laboratory rodents with no frequent contact with pathogens and/or antigens have greater immunological focus resulting in strong Th1 proinflammatory cytokine responses during viral infections [10]. Similarly, severe COVID-19 cases are associated with increase in Th1 and Th17 cell proportions as well as inflammatory CD14+CD16+ monocytes [3,19]. This has been linked to high expression of Interleukin 6 (IL-6) and Interleukin 23 (IL-23) that accelerate inflammation and promote the conversion of naïve CD4+ T cells into Th17 cells [20,21]. Consequently, high levels of Th1, Th17, and inflammatory CD14+CD16+ monocytes release more cytokines that activate macrophages and fibroblasts resulting in overwhelming amounts of proinflammatory cytokines [21,22] associated with severe lung pathology in COVID-19 patients [3].

**Oestrogen and testosterone:** overwhelming cytokine responses in older patients with COVID-19 follow this mechanism [2,3,19]. However, we think that hyper inflammation in older patients may also be related to pre-existing high levels of IL-6 and other pro-inflammatory cytokines associated with low oestrogen and testosterone levels due to menopause or andropause [23]. Oestrogen and testosterone are known inhibitors of secretion of IL-6 [23] but where low muscle mass (in the case of men) and chronic disease such as diabetes, hypertension, and heart diseases exist, the effects of oestrogen and testosterone are downgraded thereby risking an individual to cytokine storms. Thus, we believe that levels of oestrogen and testosterone may determine an individual´s susceptibility to severe COVID-19 and death. Chronic diseases and obesity do not only suppress oestrogen and testosterone but also promote high levels of pro-inflammatory cytokines making the risk of severe SARS-CoV2 infections even higher. This explains the high cases of severe COVID-19 cases and death rates in Europe and North America due to a high population of old people and those with chronic diseases. Conversely, we expect fewer cases of severe forms of this disease and deaths in most Sub-Saharan African countries where the majority of people are young but high HIV rates may dilute this effect.

**Figure 1:**
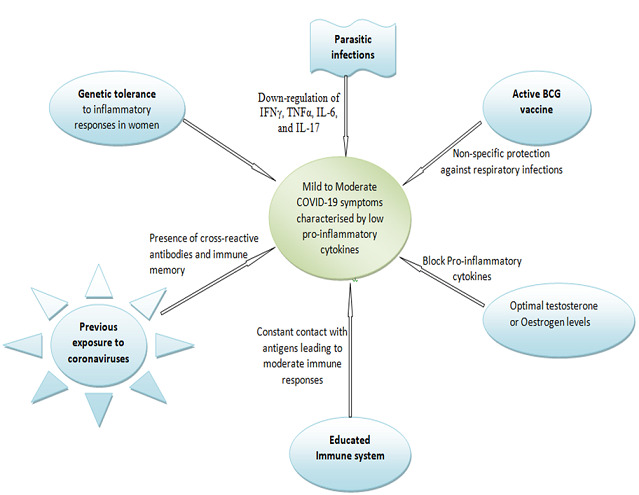
Factors and mechanisms that may contribute to mild inflammatory responses in COVID-19 leading to mild-moderate or asymptomatic

**Educated immune systems:** since proinflammatory cytokines are at the centre of the patho-mechanism of severe SARS-CoV-2 infections, we think that an educated immune system particurly in immune-competent individuals may have a role in asymptomatic COVID-19 (Figure 1). We define an educated immune system as one that has had frequent exposure to all kinds of pathogens and antigens and is evolved enough to respond without causing self-harm. Such an immune system may be common in immune-competent people living in infectious disease burdened areas and in those exposed to various respiratory pathogens in public places (schools, colleges, Universities, hospitals etc) due to close contact. Similar to what is observed in wild rodents, we expect such individuals to have immune responses to SARS-CoV-2 characterised by a balanced Th1 and Th2 cytokine response that is neither depressed nor hyperactive but none pathological. This is because daily exposure to pathogens allows the immune system to have a much less focused immune response thereby lowering its aggressiveness but maintaining its effectiveness [10,24]. However, where the individual has had exposure to parasitic infections (intestinal helminths and others), the immune responses will be skewed towards elevated Th2 responses with depressed Th1 cytokines resulting in mild immune mediated damage during virus infection such as SARS-CoV-2. Part of our conviction regarding the role of constant contact with pathogens in the environment in moderating COVID-19 progression is based on the predominant theory regarding the rise of allergic reactions brought about by improvements in vaccination and sanitation [25-27]. In both allergic reactions and infectious diseases, it appears that immune education is vital for favourable prognosis of some diseases or infections. In line with exposure theory, we think that previous exposure to some strains of coronaviruses antigenically similar to SARS-CoV-2 may also be a contributing factor to some cases of mild or asymptomatic COVId-19. We assume that cross-reactive antibodies may account for the protection. There is need to evaluate antibody cross reactivity among coronaviruses.

Parasitic infections: these pathogens up-regulate IL-4 and IL-10 cytokines resulting in increased Th2 differentiation and down-regulation of IFNγ, TNFα, IL-6, and IL-17 inflammatory responses, respectively [18,25,28]. We suppose that this immune mechanism is active in wild rodents that presumably have high parasitic infestations and may play a role in trade-off mechanisms between viruses and mice [13,17]. Thus, parasite driven down-regulation of pro-inflammatory mediators suggest the potential of these pathogens to locally and systematically block cytokine storms observed in COVID-19 cases or any other viral illness [25,28]. Taken together, we speculate that such a scenario is possible in parasite infested individuals living in resource-limited communities where regular deworming is not practised. Therefore, we envisage mild to moderate or self-limiting COVID-19 cases in such individuals as long as their immune system is competent and are free of chronic diseases.

Genetic tolerance: mortality and vulnerability data for the COVID-19 infections in China show sex differences between men and women with more men dying than women [29]. Smoking and other risky behaviours among men have been suggested as possible reasons for the observation [29]. Here we suggest the role of genetic tolerance to cytokine storms in women. IL-6, one of the cytokines at the centre of the pathology of COVID-19, is widely expressed in the female reproductive tract and gestational tissues to enable embryo implantation, placenta development, and pregnancy tolerance [30]. We speculate that immuno-competent women may be genetically prepared to handle cytokine storms than men leading lowered risk of COVID-19 associated death.

## Conclusion

The mechanism(s) for the occurrence of mild or severe COVID-19 is related to an individual´s immune system, particularly the levels of pro-inflammatory responses. Genetic tolerance related to pregnancy may also have a role to play. Parasites, sex hormones, and constant exposure to pathogens may help lower the risk of pathological immune responses in SARS CoV-2 infections in immuno-competent individuals. Since the risk of severe disease is high among the elderly and those with chronic diseases, community testing for the virus must prioritise these groups of people in the light of shortage of testing kits. This will ensure that those found positive receive appropriate medical attention before they show any symptoms.

## Competing interests

The author declares no competing interests.
